# Brain death and the apnea test

**DOI:** 10.4103/0972-2327.56327

**Published:** 2009

**Authors:** Rupa Sreedhar, Sanjeev V. Thomas

**Affiliations:** Department of Anesthesiology, Sree Chitra Tirunal Institute for Medical Sciences and Technology, Trivandrum, India; 1 Department of Neurology, Sree Chitra Tirunal Institute for Medical Sciences and Technology, Trivandrum, India

Brain death and brainstem death are two important medicolegal issues that have drawn much public interest in the recent past. Pronouncement of death has important ethical, social, psychological, and medical dimensions. In this issue of the journal we publish a technical note on the apnea test for the diagnosis of brainstem death.

We reiterate that brainstem death is a conclusion drawn on the basis of several findings. An in-depth discussion on brainstem death is beyond the scope of this commentary. The clinician is often called upon to comment on brainstem activity or inactivity in order to pronounce brainstem death. We would like to stress that the clinician should be satisfied with identifying the precise cause of coma, its irreversibility, and the demonstration of irreversible failure of all brainstem functions. The bedside tests for confirming the failure of brainstem functions include the pupillary light reflex (midbrain function); the corneal reflex, the oculocephalic reflex, and the ice-cold caloric test (pontine functions); and the gag reflex, the cough reflex, the atropine test for vagus-mediated tachycardia, and the apnea test (medullary function). These tests need to be carried out by clinicians who are not part of the treating team and organ donation / harvesting team and who have sufficient expertise in the test(s). It is important to demonstrate the irreversibility of brainstem failure by confirming the absence of these brainstem signs after an interval of 6–24 h.

The laws and guidelines pertaining to death vary from country to country and between states in some countries.[1] Each country has its own laws and rules regarding brainstem death and the procedure to be followed for confirming it. The tests need to be carried out only after satisfying the prerequisites. The standard procedures need to be carefully adopted. It is important that the family or friends of the subject are taken into confidence and their consent obtained after a detailed discussion.

A recent article has drawn attention to the various pitfalls while making a diagnosis of brain death.[1] The purpose of this technical report is to bring to the attention of the readers the intricacies of the apnea test and to familiarize them with the commonly followed techniques. The apnea test is only one of the many tests that are done to ascertain medullary function. It should be interpreted in the light of the other tests for brain death and the underlying disorder and recent medications received by the patient. We recommend that each institution set up its own guidelines (in the light of the existing laws) on when to invoke the brainstem death procedures, how to carry it out, what would be the clinical context in which it would be ordered, who would order the tests, who would carry out the tests, and how it would be documented in the records. It will be worthwhile to have occasional clinical audits of the issue and update the guidelines periodically.
